# Use of OCT Angiography to Diagnose and Manage Atypical Presentations of Macular Telangiectasia Type 2

**DOI:** 10.3390/ijms23147849

**Published:** 2022-07-16

**Authors:** John Moir, Shivam V. Amin, Saira Khanna, Rahul Komati, Lincoln T. Shaw, David Dao, Seenu M. Hariprasad, Dimitra Skondra

**Affiliations:** 1 Pritzker School of Medicine, University of Chicago, Chicago, IL 60637, USA; jmoir@uchicago.edu; 2 Department of Ophthalmology and Visual Science, University of Chicago Medical Center, Chicago, IL 60637, USA; shivam.amin@uchospitals.edu (S.V.A.); sairakhanna1092@gmail.com (S.K.); rahul.komati@gmail.com (R.K.); lincoln.shaw@uchospitals.edu (L.T.S.); dtdao1@gmail.com (D.D.); sharipra@bsd.uchicago.edu (S.M.H.); 3 Georgia Retina, Stockbridge, GA 30281, USA; 4 J. Terry Ernest Ocular Imaging Center, University of Chicago Medical Center, Chicago, IL 60637, USA

**Keywords:** optical coherence tomography angiography, imaging, retina, macular telangiectasia

## Abstract

Macular telangiectasia Type 2 (MacTel) is a bilateral acquired retinal disease characterized by both vascular changes and atrophy of the retina. The purpose of this case series is to highlight the use of optical coherence tomography angiography (OCTA) as a non-invasive imaging modality to distinguish atypical MacTel from other macular conditions with similar presentations. We performed a retrospective review of patients referred to our academic retinal practice with unconfirmed or misdiagnosed MacTel between July 2017 and July 2021. Patients’ OCTA imaging findings were reviewed to guide the appropriate diagnosis and management of atypical MacTel. Fifteen eyes from eight patients were included in this study. Six patients were referred with previous diagnoses of either full-thickness macular hole, lamellar hole, vitreomacular traction (VMT), postoperative cystoid macular edema (CME), or diabetic macular edema (DME). Two patients were referred to us to confirm the diagnosis of MacTel. OCTA revealed telangiectatic vessels in the temporal parafovea of all 15 eyes. OCTA also highlighted previously undiagnosed subretinal neovascularization (SRNV) in seven eyes. OCTA imaging is a valuable imaging modality to distinguish MacTel from other macular conditions, whose treatment courses vary substantially. Due to its ease of use, it holds immense potential in the future as treatments for non-proliferative MacTel emerge.

## 1. Introduction

Idiopathic macular telangiectasia type 2 (MacTel) is an acquired retinal disease characterized by bilateral neurodegenerative changes in the macula with subsequent telangiectatic changes to the temporal vessels of the parafovea [1]. On fundus examination, right-angled venules, crystalline deposits, reduced retinal transparency, and ectatic capillaries may be seen [2]. Retinal neurosensory atrophy of the photoreceptors occurs with disease progression, and subretinal neovascularization (SRNV) is a complication that can occur at any point during the disease course [3]. Examination findings can be subtle and often lead to the misdiagnosis of MacTel as another condition, such as age-related macular degeneration (AMD), macular hole, or diabetic macular edema (DME). While MacTel was initially considered to be primarily a microvascular condition, recent evidence supports its reclassification as a neurodegenerative condition due to the depletion of Müller glial cells of the retina [4,5]. Patients with MacTel typically present in the sixth or seventh decades of life, complaining first of difficulty reading or metamorphopsia [6]. MacTel has an estimated prevalence as low as 0.005% to 0.1% in adults over the age of 40 [7,8]. Given its low prevalence and subtle clinical examination findings, MacTel may easily go undetected or be misdiagnosed.

Multimodal imaging modalities are especially useful to help distinguish MacTel from other conditions whose clinical course and treatment may differ substantially. Traditionally, fluorescein angiography (FA) has been the gold standard for diagnosing MacTel, revealing telangiectatic vessels that leak at the temporal parafovea [9]. However, in recent years there has been a shift towards diagnosis with a combination of non-invasive imaging modalities, such as optical coherence tomography (OCT), fundus autofluorescence (AF), confocal blue reflectance imaging, and OCT angiography (OCTA) [10,11]. Characteristic OCT findings include cavitations of the inner and outer retina, draping of the internal limiting membrane (ILM), disruption of the ellipsoid zone (EZ) layer, and foveal thinning [12,13]. OCTA is a promising, non-invasive imaging modality for visualizing and monitoring vascular changes in MacTel, especially when SRNV is present. Of note, OCTA will best diagnose MacTel when pathologic vascular changes have already occurred, which does not always precede the neurodegenerative alterations also associated with MacTel [1]. In these latter cases, alternative methods such as OCT, fundus AF, or confocal blue reflectance imaging may be superior diagnostic tools.

OCTA detects moving erythrocytes and captures repeated B-scans at various positions to generate a depth-resolved map of the retinal microvasculature [14]. In contrast with FA, OCTA is non-invasive and has quick acquisition speeds, making it readily integrable into clinical practice where longitudinal follow-up is required. OCTA reveals characteristic features of MacTel, including right-angled vessel branching, telangiectatic vessels predominantly in the deep capillary plexus (DCP) at early disease stages, and SRNV in later stages [15,16,17].

Quantitative studies have begun to evaluate differences in OCT and OCTA parameters between MacTel eyes and healthy eyes. Vessel density in the superficial capillary plexus (SCP) and DCP are diminished in eyes with MacTel, with vessel reduction becoming especially pronounced at the DCP late in the disease [9,18,19,20]. MacTel eyes are also associated with a larger foveal avascular zone (FAZ) area that negatively correlates with visual acuity [18,19]. EZ loss, which is an indicator of photoreceptor impairment, has also been reported to be predictive of worsening visual function in MacTel cases, particularly when areas of EZ loss extend from the temporal macula into the central fovea and nasal macula [21,22].

In this longitudinal, observational case series of patients referred to our academic retina clinic with initially misdiagnosed or unconfirmed MacTel, we discuss the use of OCTA imaging to guide diagnosis when other macular conditions could reasonably be suspected. Additionally, we examine quantitative changes in OCTA parameters from initial presentation to follow-up.

## 2. Results

### 2.1. Patient Demographics

Fifteen eyes of eight patients with MacTel were identified and included in this study. A clinical summary of each of these patients is available in Table 1. The right eye of patient G was excluded as it was not imaged at initial presentation. Patients presented for an initial consultation at a mean age of 65.8 ± 6.6 years (range, 54–74). Five patients (62.5%) were male. Six patients (75%) identified as Caucasian and two (25%) identified as African-American. The average follow-up time was 1.6 ± 1.2 years (range, 0.3–3.8 years). Mean best-corrected visual acuity (BCVA) (LogMAR) of all patients at baseline presentation was 0.33 ± 0.25 and 0.25 ± 0.18 at their last visit (approximate mean Snellen equivalent = 20/43 and 20/36, respectively). There was a statistically significant improvement from baseline to final BCVA (*p* = 0.035).

### 2.2. Referring Diagnoses and OCTA Findings

Of these eight patients, two were referred to us to confirm a MacTel diagnosis, while six were referred with other suspected conditions. These conditions included one patient with DME, four patients with macular holes, one patient with a lamellar hole, one patient with vitreomacular traction, and one patient with postoperative cystoid macular edema (CME). OCT and OCTA imaging revealed telangiectatic vessels in the parafovea of the DCP, cavitary changes, and EZ disruption in all 15 eyes (100%), confirming the diagnosis of MacTel.

**Figure 1:**
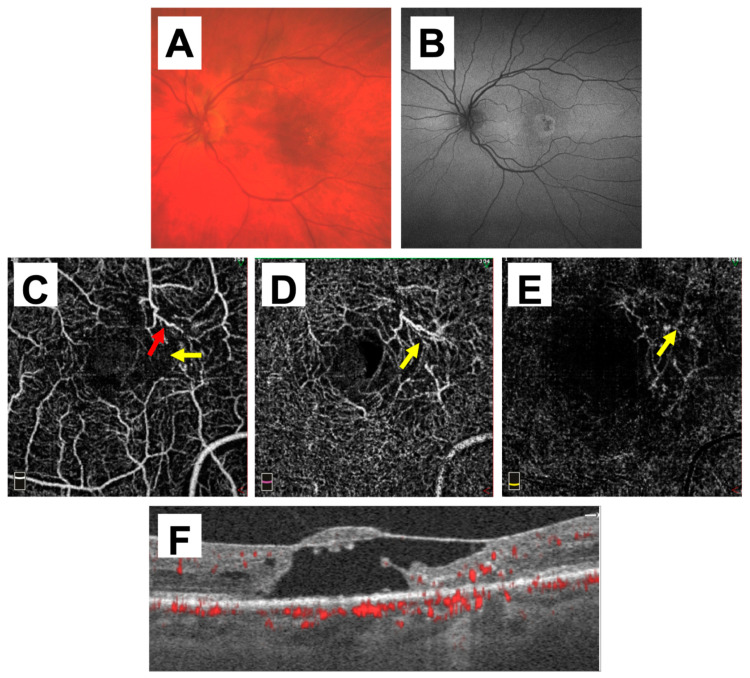
Multimodal imaging findings in Patient F, who was initially diagnosed with bilateral macular holes. Left eye is shown as a representative example. (**A**) Fundus pseudo-color image reveals subtle hyperpigmentation and atrophy concentrated in the temporal macula. (**B**) Fundus autofluorescence (AF) reveals a corresponding, well-delineated area of increased autofluorescence with a central area of decreased autofluorescence. (**C**) Imaging of OCTA superficial capillary plexus (SCP) reveals evidence of mild vessel changes (red arrow) and dropout (yellow arrow) concentrated temporally. (**D**) Deep capillary plexus (DCP) features parafoveal telangiectasias and vessel dilation, primarily focused in the temporal hemifield (yellow arrow). (**E**) Evident in the outer retina is neovascularization (yellow arrow), which can be seen to arise from the deep retinal layers. (**F**) OCT B-scan through the central fovea reveals cystic changes, which can masquerade and be misinterpreted as a macular hole without accompanying OCTA vessel map.

Macular or lamellar holes were common misdiagnoses, appearing on the referral for nine eyes and including two eyes of one patient who had concurrent MacTel and bilateral macular holes treated with drop therapy (Table 1: Patient B). DME or postoperative CME were noted on the referral for three eyes. Patient F is an example of an OCT B-scan that could readily be misinterpreted as a full-thickness macular hole with an overlying ILM drape (Figure 1). However, the accompanying OCTA vessel map clearly reveals vascular abnormalities indicative of MacTel in the parafovea of the DCP, with mild vessel changes in the SCP and neovascularization in the outer retina.

**Figure 2:**
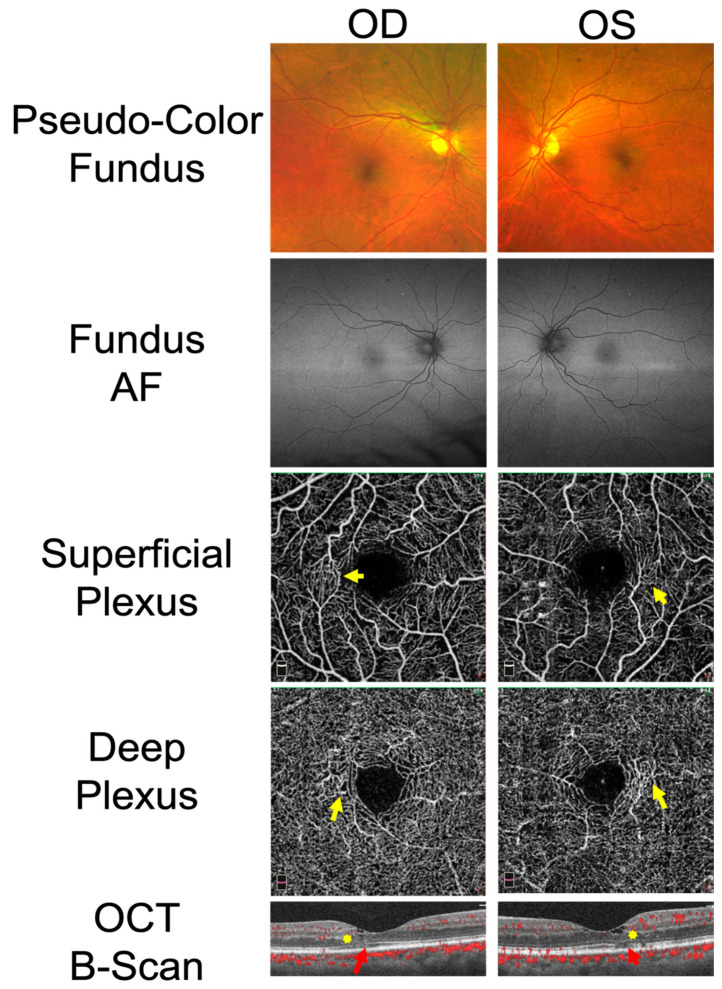
Multimodal imaging findings in patient H, who presented for a diabetic eye evaluation concerning diabetic macular edema (DME). Right and left eyes are shown. Pseudo-color fundus and fundus autofluorescence (AF) imaging are unremarkable. In both eyes, imaging of the superficial and deep capillary plexuses reveals telangiectatic vessels, primarily concentrated temporally in the superficial plexus, but becoming circumferential in the deep plexus (yellow arrows). OCT B-Scan shows minor cystic changes occurring in the inner retina and focally limited to the temporal fovea (yellow stars). Ellipsoid zone (EZ) loss is also present predominantly temporally to the fovea (red arrows).

Patient H is an example of an early-stage MacTel presentation that was initially misdiagnosed as DME. While minor cystic changes were present temporally in the fovea of the inner retina, a closer review of OCTA B-scans unveiled areas of EZ loss temporally and uncovered telangiectatic vessels primarily concentrated in the temporal parafovea (Figure 2). Of note, color fundus and fundus AF imaging were unremarkable. However, the Optos system uses green light fundus autofluorescence, which is not absorbed by macular pigment. Hence, loss of macular pigment in MacTel cannot be visualized.

### 2.3. Management of Eyes with SRNV

**Figure 3:**
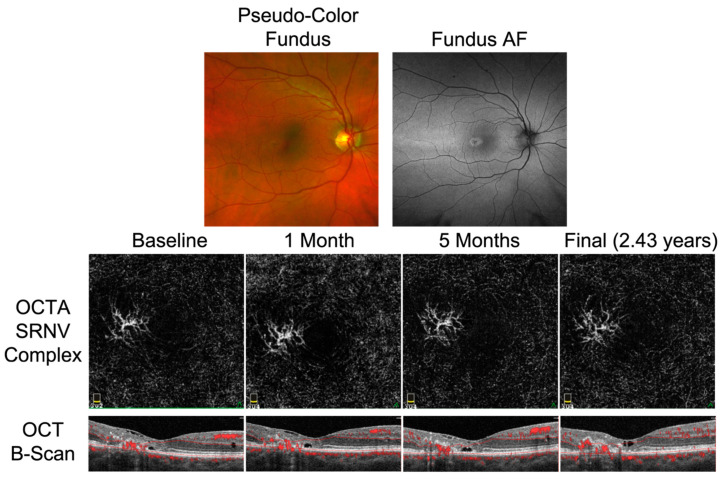
Response of subretinal neovascularization (SRNV) to treatment, as tracked by OCTA imaging of the outer retina. Baseline pseudo-color fundus and fundus autofluorescence (AF) imaging shown for reference, demonstrating a secondary neovascular membrane in the temporal macula. The right eye of patient D was treated with intravitreal bevacizumab at 1 and 5 months after baseline presentation. The SRNV complex, located in the temporal aspect of the outer retina, displays stability with respect to its size and location during treatment and continued monitoring. OCT B-scans through the central fovea shown at bottom, with overlying flow in red.

SRNV was observed in seven eyes (46.67%). Two of these eyes (28.6%) were treated with an intravitreal anti-vascular endothelial growth factor (VEGF) injection at least once, while the other five eyes (71.4%) were observed over the entirety of the follow-up period. BCVA of one anti-VEGF treated eye improved from 20/125 at baseline to 20/60 after three injections of bevacizumab (Table 1: Patient E, right eye). BCVA of the other anti-VEGF treated eye also improved from 20/60 to 20/40 after two injections of bevacizumab (Table 1: Patient D, right eye) and the SRNV remained stable in follow-up OCTA images (Figure 3). SRNV was not part of the referring diagnosis of patients sent to our clinic by outside providers and was newly diagnosed after OCTA imaging in all seven eyes.

### 2.4. Treatment of Co-Existing Macular Holes

**Figure 4:**
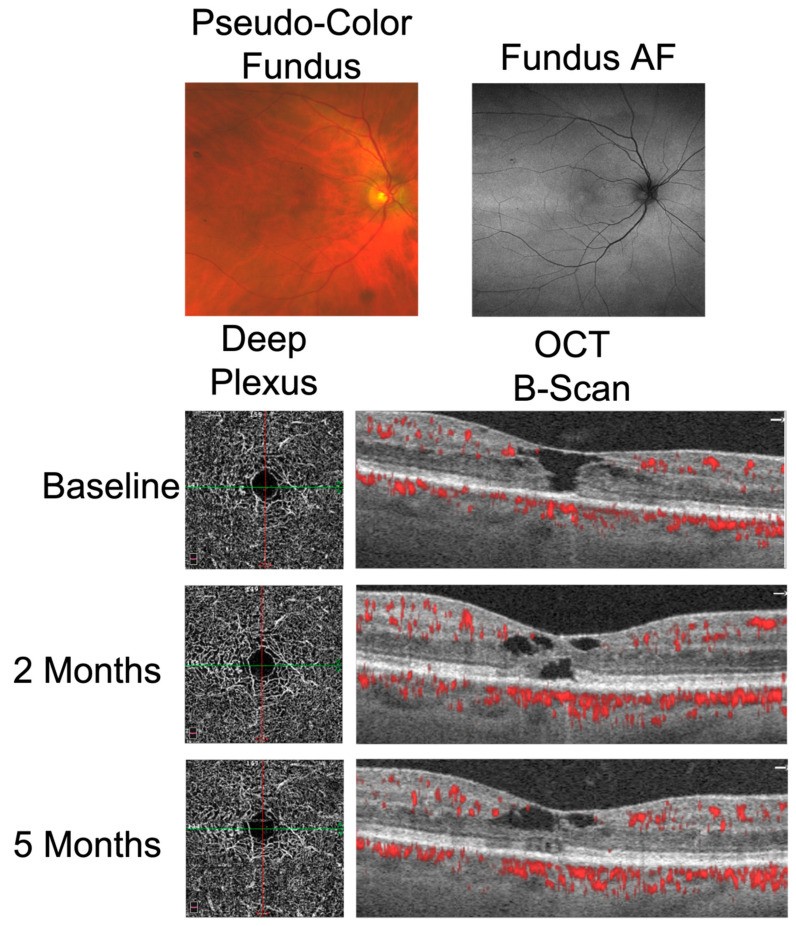
Closure of the outer retinal layers of the right eye following drop therapy in patient B with MacTel. Pseudo-color fundus imaging demonstrates subtle hypopigmentation in the temporal parafovea and fundus autofluorescence (AF) reveals a corresponding area of increased autofluorescence. OCTA of the deep capillary plexus is shown at left, with B-scans shown at right. OCT B-Scans are taken through the central fovea, as shown by the green line through the deep capillary plexus vessel map. Patient B followed a drop therapy regimen consisting of prednisolone acetate four times per day, ketorolac four times per day, and dorzolamide three times per day for seven months in the right eye. Cavitations of the inner retina persisted after 8 months of drop therapy.

Patient B presented with bilateral, full-thickness macular holes with concurrent MacTel. While the initial macular hole diagnosis was correct, it was incomplete. OCTA imaging revealed the classic dilated vessels of MacTel, extending circumferentially around the fovea of the DCP (Figure 4), thereby facilitating the proper diagnosis of MacTel with a concurrent macular hole.

Patient B was treated for a concurrent full-thickness macular hole in the right eye and a stage I macular hole in the left eye using a drop therapy regimen, consisting of topical prednisolone acetate 1% four times per day and ketorolac 0.5% four times per day, followed for seven months in the right eye and three months in the left eye, respectively, at the time of this study. The right eye also received topical dorzolamide 2% three times per day. Following drop therapy, the BCVA of the right eye improved from 20/60 at baseline to 20/40 and the full-thickness macular hole closed in the inner retinal layers (Figure 4). The BCVA of the left eye remained stable at 20/30, while the stage I macular hole decreased in size.

### 2.5. OCTA Quantitative Parameters

Quantitative OCTA parameters were available at baseline and final follow-up for 12 eyes from eight patients. For the other three eyes, comparison between baseline and final OCTA parameters was not possible due to poor quality follow-up OCTA images that did not meet inclusion criteria. We used a paired *t*-test to compare baseline and final vessel density at the SCP and DCP, FAZ area, acircularity index (AI), and FD-300, along with the length of EZ loss. No significant differences were found between any of these parameters between baseline and final follow-up (Table 2).

## 3. Discussion

MacTel is a bilateral, acquired disease characterized by neurodegeneration and subsequent vascular ectasia, with other vascular alterations including SRNV arising from the deep retinal vascular layers [1,2,3]. In recent years, there has been a shift away from diagnosis with FA to other methods such as OCT, fundus AF, and confocal blue reflectance imaging. Fundus AF offers a high diagnostic performance for the detection of MacTel [10]. OCTA is another promising imaging modality for the clinical management of MacTel, providing non-invasive and rapid imaging. Combined with OCT scans that capture neurodegenerative changes in MacTel, such as EZ disruption and cystic cavities, OCTA displays characteristic vascular patterns, including telangiectatic vessels and SRNV.

Use of OCTA in this series revealed accompanying telangiectasias within the temporal parafovea of the DCP in all patients misdiagnosed with macular/lamellar holes or macular edema, allowing for the correct diagnosis of MacTel (Figure 1 and Figure 2). Given that macular holes and macular edema can display similar cavitary-like lesions and intraretinal cysts on OCT, combined with the overall rarity of MacTel, such findings alone may not immediately point towards a MacTel diagnosis, especially when multimodal imaging is unremarkable. In these cases, OCTA can be a useful aid in proper diagnosis [23].

Furthermore, MacTel can present concomitantly with other macular diseases, which may warrant additional or supplementary treatment. An association between macular or lamellar holes and MacTel has been well-characterized [24]. The standard of care treatment for full-thickness macular holes is pars plana vitrectomy (PPV) with and without gas tamponade or internal limiting membrane peeling. This surgical intervention requires several days of face-down positioning post-operatively. The few studies that have evaluated the effectiveness of PPV have noted no significant differences in visual acuity between MacTel eyes with macular holes treated surgically compared to those that were medically managed without surgery [25], lower rates of macular hole closure with PPV in MacTel eyes compared to idiopathic macular holes without MacTel also treated surgically [26], and overall limited reports of successful macular hole closure in a number of eyes with MacTel [27,28]. Hence, surgical intervention of concomitant macular holes with MacTel may not be the preferred treatment for all patients.

Recently, Sokol et al. described a case series of patients with idiopathic macular holes who were treated using non-invasive drop therapy consisting of a topical steroid, a non-steroidal anti-inflammatory agent, and a carbonic anhydrase inhibitor [29]. Patient B received a drop therapy regimen as described above for seven months in the right eye, resulting in an improvement in visual acuity from 20/60 to 20/40. At five months, closure of the outer retina was noted, while cavitations of the inner retina persisted (Figure 4). These cavitations of the inner retina persisted at the time of this report, of approximately eight months’ duration. The left eye also received a drop therapy regimen for three months. While improvement was noted on OCT in the outer retina after three months, tissue loss of the inner retina developed. However, at this time, the patient had not completed the full drop-therapy treatment course. It is possible that closure of the macular hole in the right eye occurred spontaneously, as has been reported in other patients with MacTel [30,31,32]. This case highlights a potential alternative therapy in patients with MacTel who desire to avoid the invasiveness and post-operative discomfort associated with PPV for macular holes.

Securing an early-stage diagnosis may prove to be especially important in the coming years, as treatments for the non-proliferative stages of MacTel are made commercially available. Stage 1 and 2 clinical trials for a ciliary neurotrophic factor (CNTF) implant demonstrated safety and a slowing of neuroretinal degeneration, as measured by the area of ellipsoid zone loss compared to a sham control [33,34]. The neuroprotective effects of CNTF will likely be most pronounced when administered in the earliest stages of MacTel, before extensive photoreceptor loss and SRNV have occurred. An early MacTel diagnosis is now more important than ever, and further study of OCTA’s ability to identify patients that are eligible for currently ongoing CNTF clinical trials and those patients that will benefit from these neuroprotective agents in the near future is warranted.

This case series also highlights the power of OCTA to detect SRNV in patients with MacTel, which may otherwise be missed with OCT B-scans alone or FA. A previous study by Tzaridis et al. highlighted that OCTA identifies neovascularization in patients with MacTel earlier than OCT B-scans, thereby allowing for earlier treatment initiation and improving functional outcomes [35]. Numerous studies have found anti-VEGF agents to be successful treatments for proliferative MacTel based on their ability to stabilize or even improve BCVA [36,37]. Figure 3 demonstrates the use of OCTA to track SRNV in response to treatment with bevacizumab. OCTA of the outer retina displays stability of the SRNV complex with respect to its size and location at the temporal parafovea. This stability was associated with a modest improvement in BCVA from 20/60 to 20/40. Five other eyes in this case series with SRNV were observed without treatment and BCVA stayed stable in these cases. OCTA can be just as useful in these cases where routine monitoring is required but treatment is not warranted.

Notably, we found that there were no significant changes in any quantitative OCTA parameter from baseline presentation to final follow-up appointment. However, we may not have been powered to detect changes in these parameters due to limited patient numbers (12 eyes). Furthermore, we grouped eyes that were observed without treatment and those that were treated with either drop therapy or anti-VEGF injections. Further prospective studies should evaluate the effect of these interventions on OCTA measurements.

The usage of OCTA in this series revealed accompanying telangiectasias within the temporal parafovea of the DCP in all patients initially misdiagnosed with either macular/lamellar holes or macular edema, allowing for the correct diagnosis of MacTel. Furthermore, SRNV from MacTel was newly diagnosed in seven eyes and was not noted prior to presentation. These are critical points, as the correct diagnosis of MacTel avoids lengthy and inappropriate treatment with intravitreal injections of anti-VEGF agents or corticosteroids [38,39]. OCT and OCTA scans can also identify MacTel presenting with a concurrent macular hole, which may have important implications regarding treatment options. Furthermore, OCTA can differentiate proliferative MacTel from exudative AMD, as both may present with neovascularization. The vascular changes in MacTel evident on OCTA imaging can effectively differentiate all these masquerading conditions and detect subtle, early changes from MacTel. OCTA is a valuable imaging modality displaying the pathognomonic vascular changes in MacTel, while also tracking SRNV, which is not discernible on color fundus or fundus AF imaging. Given its overall ease of use, OCTA holds immense potential for the coming years, especially as treatments for early stages of MacTel are made available.

## 4. Materials and Methods

This observational longitudinal study was approved by the Institutional Review Board of the University of Chicago (IRB #21-1066). All study protocols adhered to the tenets of the Declaration of Helsinki. The study conformed to the Health Insurance Portability and Accountability Act (HIPAA) of 1996 regulations. The study included patients with a confirmed diagnosis of idiopathic macular telangiectasia Type 2 followed at the University of Chicago Ophthalmology Clinic between 1 July 2017 and 1 July 2021.

All patients underwent a full ophthalmological examination, including fundus examination, measurements of BCVA, fundus pseudo-color and fundus AF imaging with an Optos device (Optos Inc, Marlborough, MA, USA), and OCTA imaging using the Optovue RTVue XR Avanti (Optovue Inc, Fremont, CA, USA, 2018.1.8.63). Relevant patient demographics and clinical information including age, race, gender, referring diagnosis, and treatment course were collected from a review of the patient’s electronic medical record.

Patients underwent OCTA imaging with the Optovue RTVue XR Avanti (Optovue Inc, Fremont, CA, USA, 2018.1.8.63) with phase 7 AngioVue software. This machine has an A-scan rate of 70,000 scans per second. Images were taken using an 840 nm light source and a 45 nm bandwidth. Two consecutive B-scans, composed of 304 A-scans each, were acquired in a 3 × 3 mm^2^ region centered on the fovea. The software included the 3D Projection Artifact Removal (PAR) Software. Only images with a signal strength index greater than 50 and a quality index greater than or equal to 7 were included in the analysis. Images with significant motion or shadow artifacts were also excluded.

We used built-in Angio-Analytics software to segment the SCP and DCP. The SCP was segmented from the ILM to 10 μm above the inner plexiform layer (IPL). The DCP was segmented from 10 μm above the IPL to 10 μm below the outer plexiform layer (OPL). Built-in software was used to measure vessel density of the parafovea at the SCP and DCP. FAZ parameters, including area, AI, and FD-300, were recorded using built-in software. FD-300 is a measurement of vessel density from the ILM to the OPL in a 300 μm ring around the FAZ. These measurements were recorded at baseline visits for all patients and at final follow-up.

**Figure 5:**
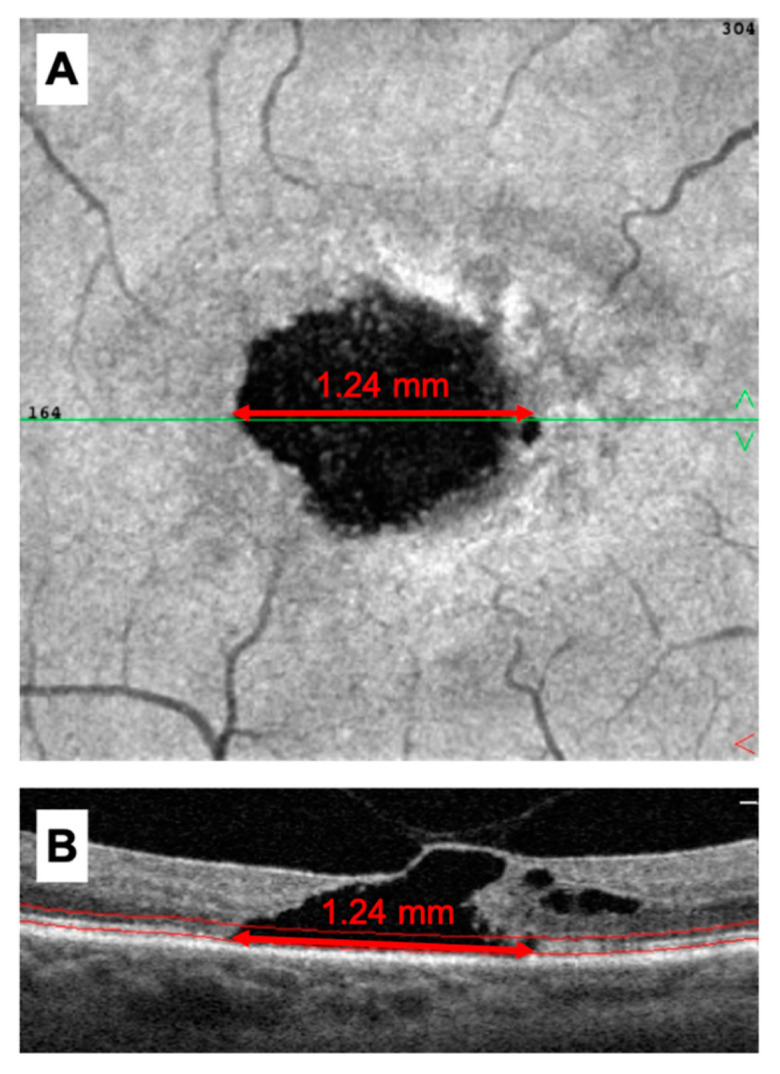
Measurement of ellipsoid zone (EZ) loss at the central fovea. (**A**) Dark or hyporeflective areas on *en*-*face* OCT represented EZ loss and was measured using the built-in caliper tool. Green line overlying *en*-*face* OCT represents the cross-sectional scan through the central fovea. (**B**) Length of EZ loss on cross-sectional OCT was cross-referenced to ensure consistency with *en*-*face* OCT. Manual segmentation of the EZ was performed as depicted by red borders on cross-sectional OCT.

The built-in caliper tool was used to measure the length (μm) of EZ loss on accompanying cross-sectional OCT B-scans traversing through the central fovea. EZ loss was defined as a clear break of the EZ layer, as opposed to areas of mere attenuation. Manual segmentation of the EZ on cross-sectional OCT allowed for *en*-*face* OCT visualization of the EZ. EZ loss on *en*-*face* images, revealed as hyporeflective or dark areas, was compared to EZ loss on cross-sectional OCT to confirm overlay (Figure 5). Measurements of EZ loss were performed for all patients at baseline and at final follow-up.

A paired sample *t*-test was used to compare baseline and follow-up OCTA parameters in all patients where those measurements were available. Statistical analyses were performed using R statistical software version 4.1.0 (The R Foundation for Statistical Computing, Vienna, Austria) within RStudio statistical software version 1.4.1717 (RStudio, Boston, MA, USA). Significance was set at *p* < 0.05 for all statistical tests.

## Figures and Tables

**Table 1:** Demographic and Clinical Information.

Patient	Demographics	Duration of Follow-Up (Years)	Eye	Referral Diagnosis	Baseline BCVA (Snellen)	Final BCVA (Snellen)	Evidence of SRNV on OCTA	Treatment
A	66-year-old Caucasian Female	0.70	OD	Unconfirmed MacTel	20/40	20/40	Yes	Observation
			OS	Unconfirmed MacTel	20/25	20/25	No	Observation
B	74-year-old Caucasian Female	0.61	OD	Macular hole	20/60	20/40	No	Drop therapy for macular hole closure
			OS	Macular hole	20/30	20/30	No	Drop therapy for macular hole closure
C	63-year-old African American Female	0.46	OD	Unconfirmed MacTel	20/20	20/20	Yes	Observation
			OS	Unconfirmed MacTel	20/20	20/25	Yes	Observation
D	70-year-old Caucasian Male	2.43	OD	Macular hole	20/60	20/40	Yes	Anti-VEGF injections at 1 and 5 months
			OS	Macular hole	20/30	20/30	Yes	Observation
E	54-year-old Caucasian Male	3.83	OD	Macular hole	20/125	20/60	Yes	Anti-VEGF injections at baseline, 1, and 3 months
			OS	Lamellar hole	20/25	20/25	No	Observation
F	74-year-old Caucasian Female	1.77	OD	VMT, macular hole	20/70	20/80	No	Observation
			OS	VMT, lamellar hole	20/80	20/70	Yes	Observation
G	64-year-old Caucasian Male	0.32	OS	Post-operative CME, cystic lamellar hole	20/100	20/50	No	Observation
H	58-year-old African American Female	2.96	OD	DME	20/30	20/25	No	Observation
			OS	DME	20/40	20/25	No	Observation

Abbreviations: BCVA, best-corrected visual acuity; SRNV, subretinal neovascularization; OCTA, optical coherence tomography angiography; OD, right eye; OS, left eye; VMT, vitreomacular traction; CME, cystoid macular edema; DME, diabetic macular edema; VEGF, vascular endothelial growth factor.

**Table 2:** Differences in Baseline and Final OCTA Parameters *.

	Baseline (SD)	Final (SD)	*p*-Value ^a^
**BCVA** (**LogMAR**)	0.33 (0.25)	0.25 (0.18)	0.035 *
**Signal Strength**	66.9 (6.26)	67.05 (5.34)	0.94
**EZ loss** (**mm**)	0.90 (0.48)	0.82 (0.68)	0.70
**Superficial** **Parafoveal VD (%)**	47.47 (2.12)	46.41 (3.17)	0.26
**Deep** **Parafoveal VD (%)**	54.71 (3.79)	54.93 (4.15)	0.86
**FAZ Area** (**mm^2^**)	0.38 (0.14)	0.37 (0.17)	0.75
**FAZ AI**	1.16 (0.05)	1.14 (0.05)	0.4
**FD-300** (**%**)	51.41 (5.29)	49.05 (8.27)	0.12

* Comparison of baseline and final OCTA parameters shown is from 12 eyes for which data were available. Abbreviations: OCTA, optical coherence tomography angiography; SD, standard deviation; BCVA, best-corrected visual acuity; EZ, ellipsoid zone; VD, vessel density; FAZ, foveal avascular zone; AI, acircularity index. ^a^ Paired Sample *t*-test.

## Data Availability

The data presented in this study are available upon request from the corresponding author.
